# Supplementary material for: Alcohol consumption and telomere length: Mendelian randomization clarifies alcohol’s effects
Source: Mol Psychiatry. 2022 Jul 26;27(10):4001–8. doi: 10.1038/s41380-022-01690-9 (PMC9718662; doi:10.1038/s41380-022-01690-9)
Supplement: Supplementary file 1 — SFigures [file 41380_2022_1690_MOESM1_ESM.docx]

**Supplementary methods**

Alcohol use disorder (AUD) cases were defined by the presence of a relevant ICD-9 (303, 30301, 30302, 30303, 3039, 30391, 30392, 30393) or ICD-10 (F102, F1021, F1022, F1023, F1024, F1025, F1026, F1027, F1028, F1029) code.

Non-linear MR: A meta-regression was performed of the linear MR estimates in each stratum against mean alcohol consumption in that stratum. Then the fractional polynomial method was used to assess whether a non-linear model fitted this meta-regression better than a linear model [27].

**Supplementary results**


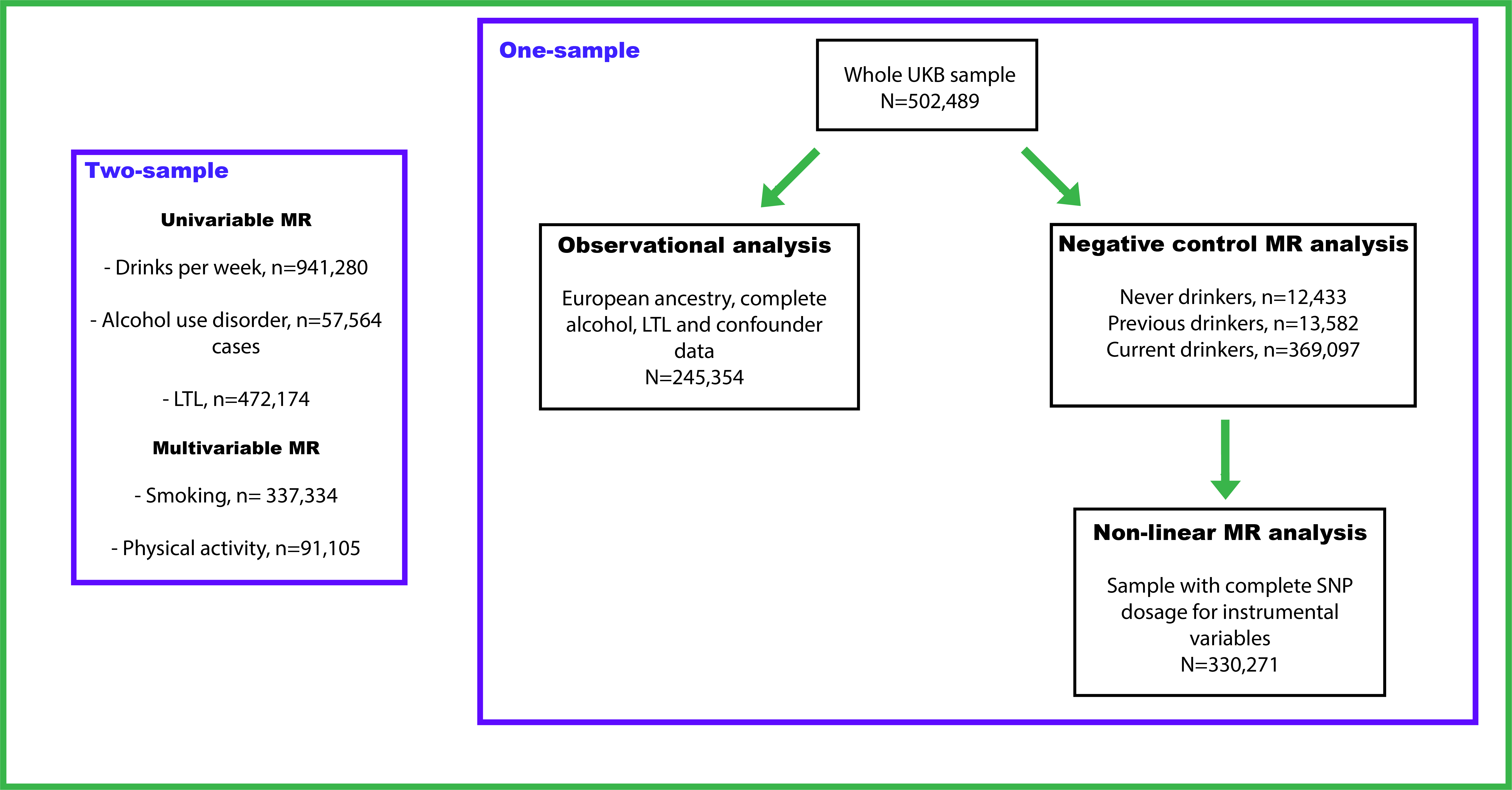


**SFigure 1: Flow chart of samples used in analyses.**

**
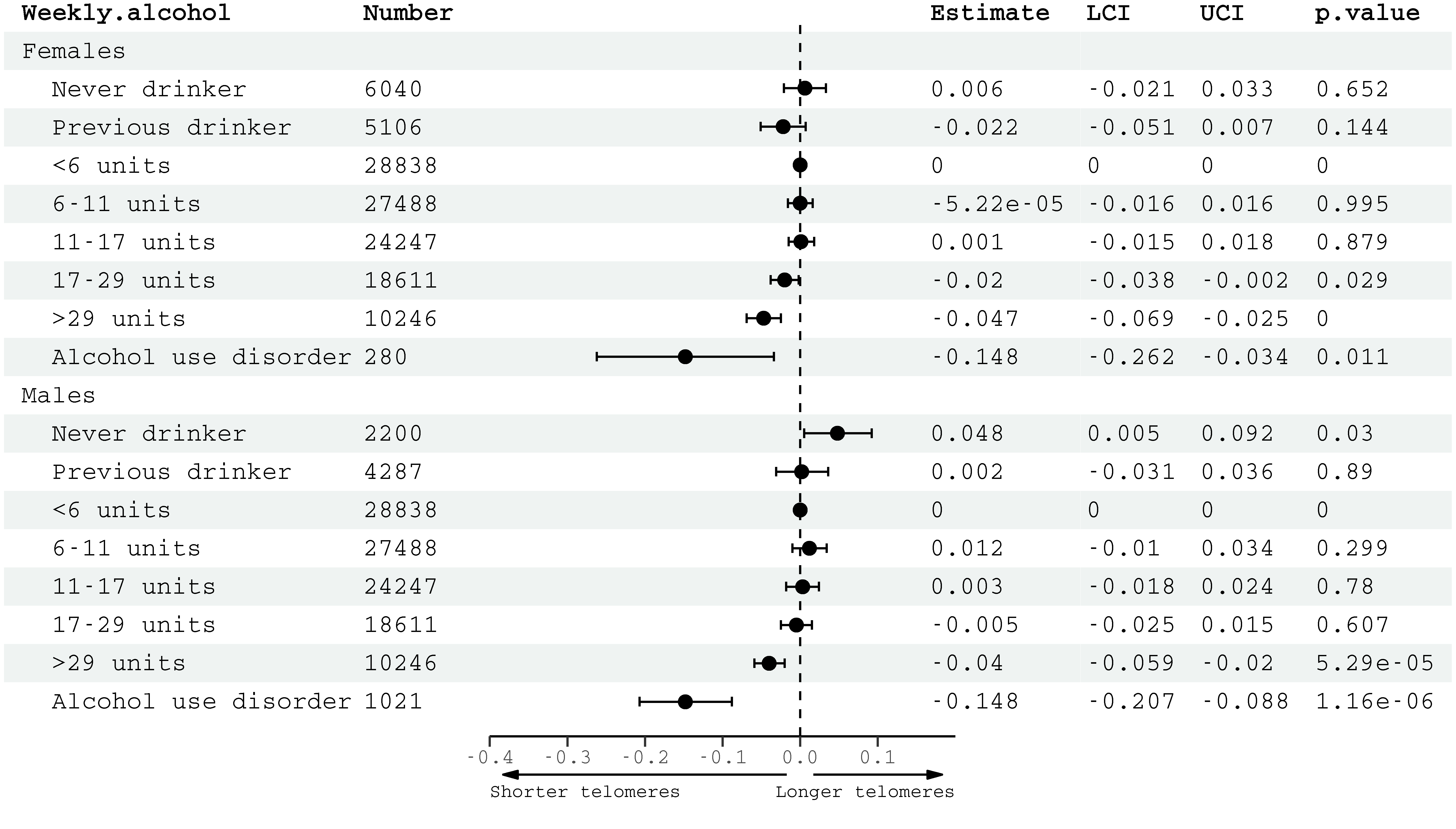
**

**SFigure 2: Observational associations with leucocyte telomere length in n=245,354 UK Biobank participants by sex. Estimates generated from two regression models: 1) alcohol intake (estimates represent SD change in LTL) and 2) ICD diagnosis of alcohol use disorder, plotted together for comparison. Reference category for alcohol intake is <6 units weekly. Models adjusted for: age, educational qualifications, leucocyte count, smoking, exercise.**


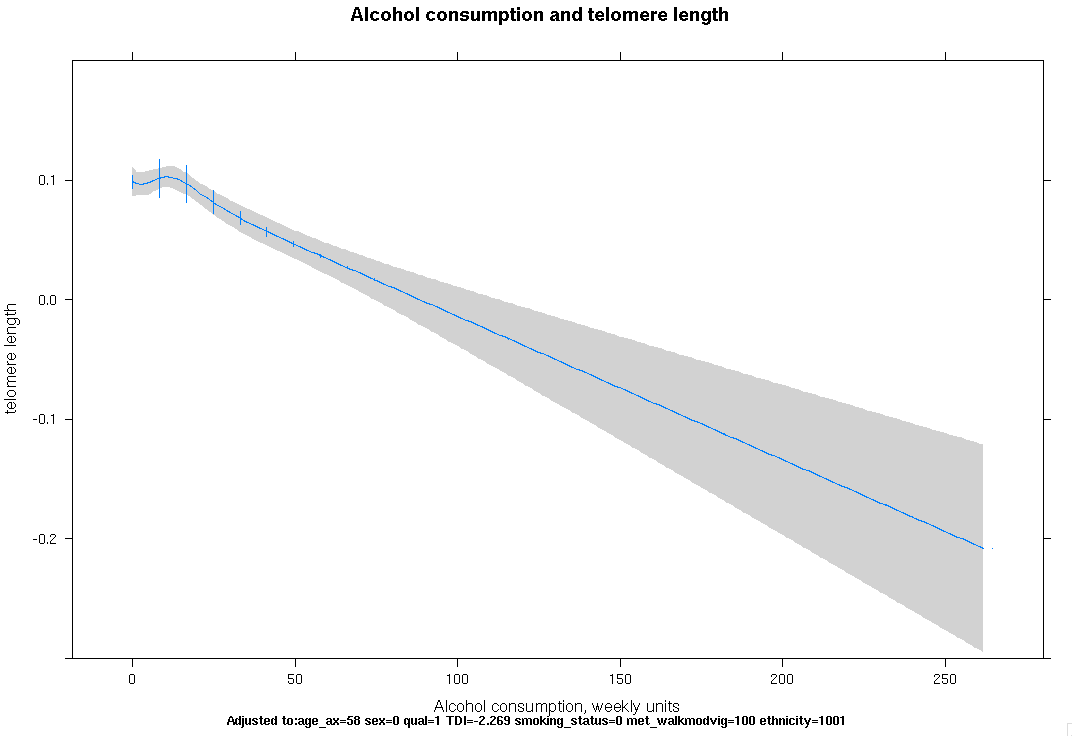


**SFigure 3: Observational association of alcohol consumption with telomere length in n=245,354 UK Biobank participants. Restricted cubic splines fitted to alcohol with 5 knots. Regression models adjusted for: age, sex, educational qualifications, smoking, leucocytes, exercise.**

**SFigure 4: Forest plot showing Mendelian randomization estimates for association of genetically-predicted alcohol consumption and telomere length for each SNP.**

**SFigure 5: Leave one out plot for genetically-predicted alcohol consumption association with telomere length.**

**SFigure 6: Funnel plot of genetically-predicted alcohol consumption association with telomere length.**

**SFigure 7: Forest plot showing Mendelian randomization estimates for genetically-predicted alcohol use disorder association with telomere length for each SNP.**

**SFigure 8: Leave one out plot for genetically-predicted alcohol use disorder association with telomere length**

**SFigure 9: Funnel plot for association of genetically-predicted alcohol use disorder and telomere length.**

IVW beta= 0.006, p=0.7

MR-Egger beta=-0.02, p=0.3

Weighted mode beta=-0.006, p=0.7

**SFigure 10: Two sample Mendelian randomization showing association of genetically-predicted telomere length and alcohol consumption (checking for reverse causation). Graph shows the strength of association between alcohol consumption and telomere SNPs on the y-axis against the telomere associations from previous genome-wide association studies for each SNP on the x-axis. A non-zero gradient to the lines indicates evidence for causality of telomere length on alcohol consumption. 85 SNPs included as instruments with complete outcome associations (n=5 SNPs palindromic with intermediate allele frequencies excluded).**

**SFigure 11: Forest plot showing estimates for genetically-predicted telomere length and alcohol consumption associations for each SNP.**

**SFigure 12: Scatterplot showing genetically-predicted telomere length and alcohol use disorder associations. N=67 SNPs with available outcome data (n=1 palindromic SNPs with intermediate allele frequencies excluded).**

**SFigure 13: Forest plot showing SNP estimates for associations between genetically-predicted telomere length and alcohol use disorder.**

| **Non-linearity test** | **P value** |
| --- | --- |
| Fractional polynomial degree | 0.9 |
| Fractional polynomial non-linearity | 0.1 |
| Quadratic | 0.1 |
| Cochran Q | 0.5 |

**STable 2: Non-linearity tests for non-linear Mendelian randomization. The best-fitting polynomial of degree 1 for the relationship between alcohol and telomere length had power 2.**

**
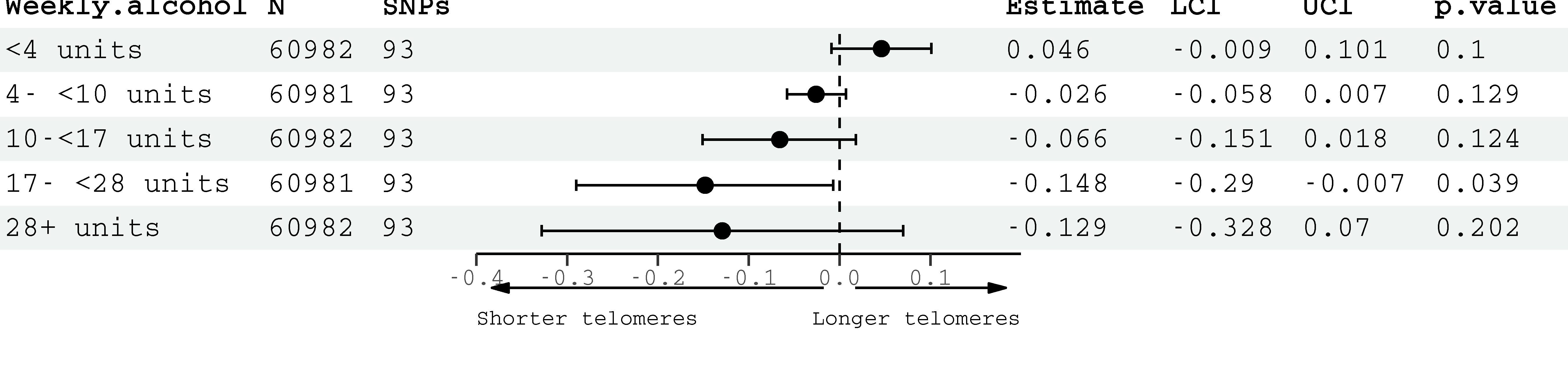
**

**SFigure 14: Non-linear Mendelian randomization showing associations between genetically-predicted alcohol consumption and telomere length, stratified by weekly alcohol intake (IV-free exposure) amongst unrelated participants only (defined as kinship coefficient <0.088).**

**
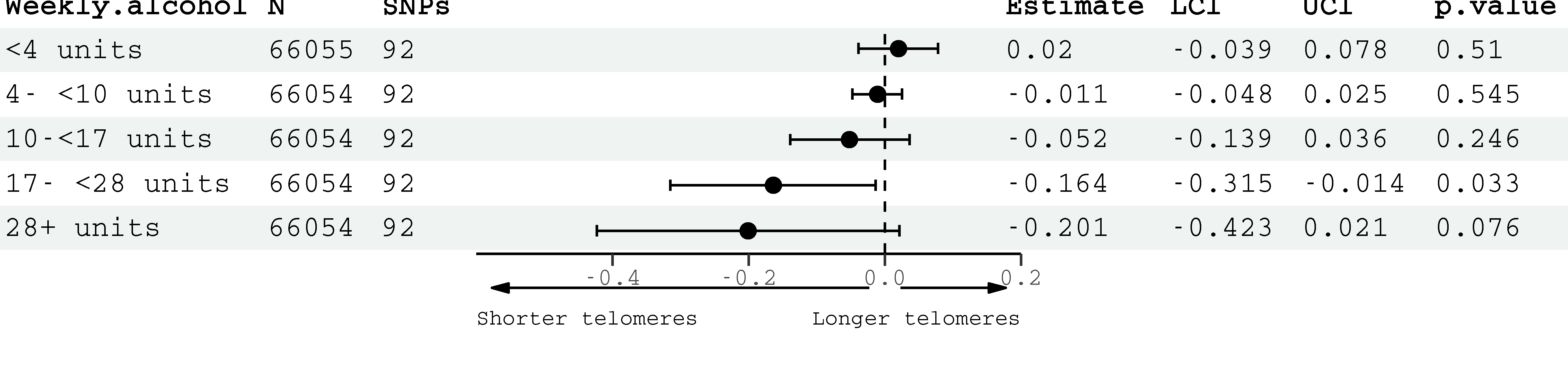
**

**SFigure 15: Non-linear Mendelian randomization showing associations between genetically-predicted alcohol consumption and telomere length, stratified by weekly alcohol intake (IV-free exposure) excluding SNP rs1229984.**

**
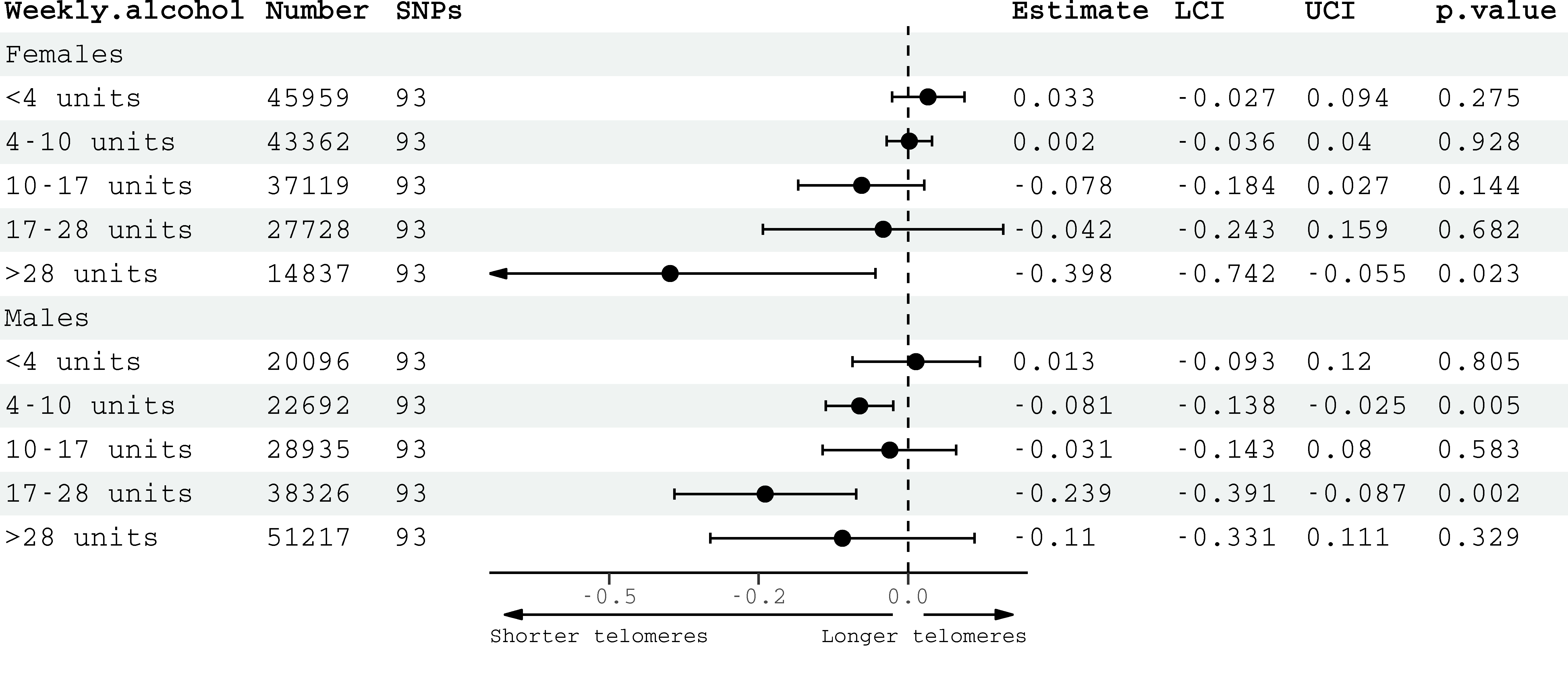
**

**SFigure 16: Non-linear Mendelian randomization showing associations between genetically-predicted alcohol consumption and telomere length, stratified by weekly alcohol intake (IV-free exposure) by sex.**

**
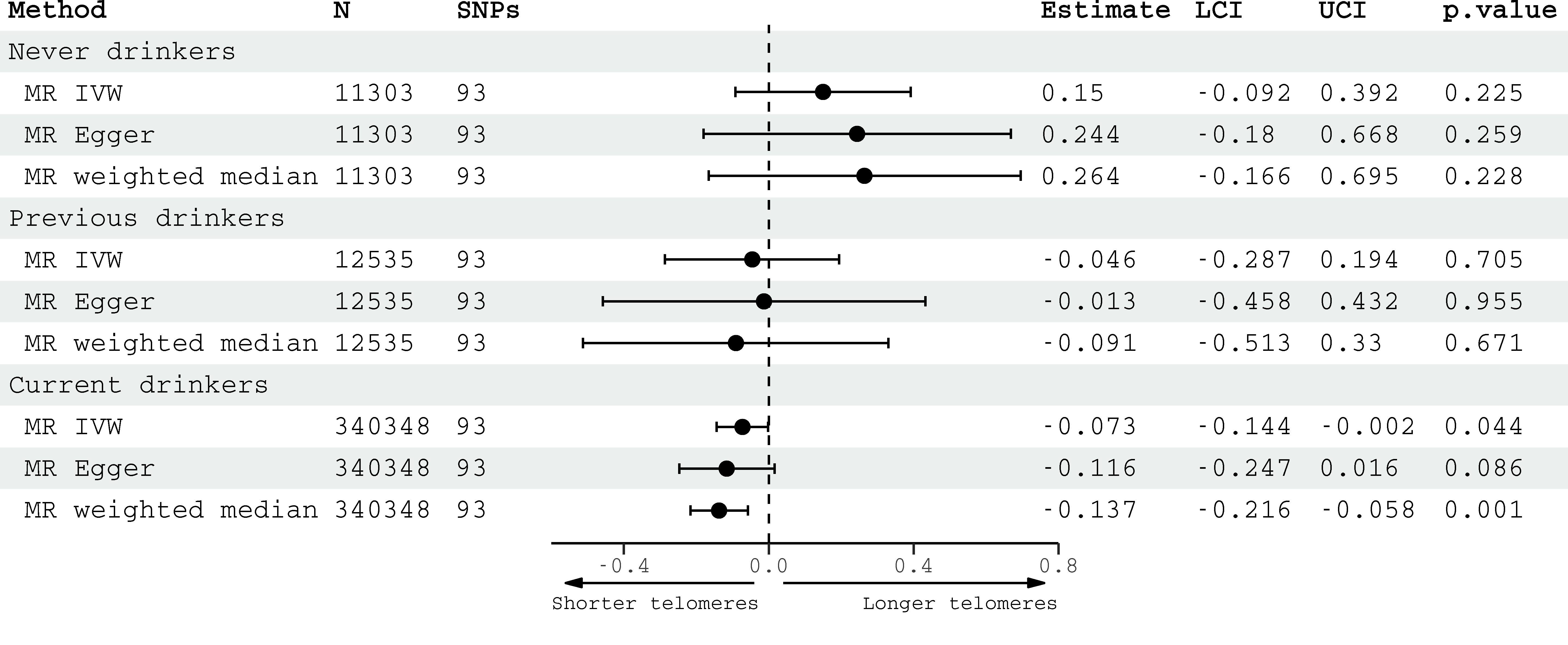
**

**SFigure 17: Negative controls for alcohol consumption, excluding related participants (kinship coefficient >0.088). Causal estimates for alcohol consumption on telomere length generated by Mendelian randomization analyses according to alcohol status. Effect estimates are per standard deviation increase in genetically-predicted log-transformed alcoholic drinks per week.**

**
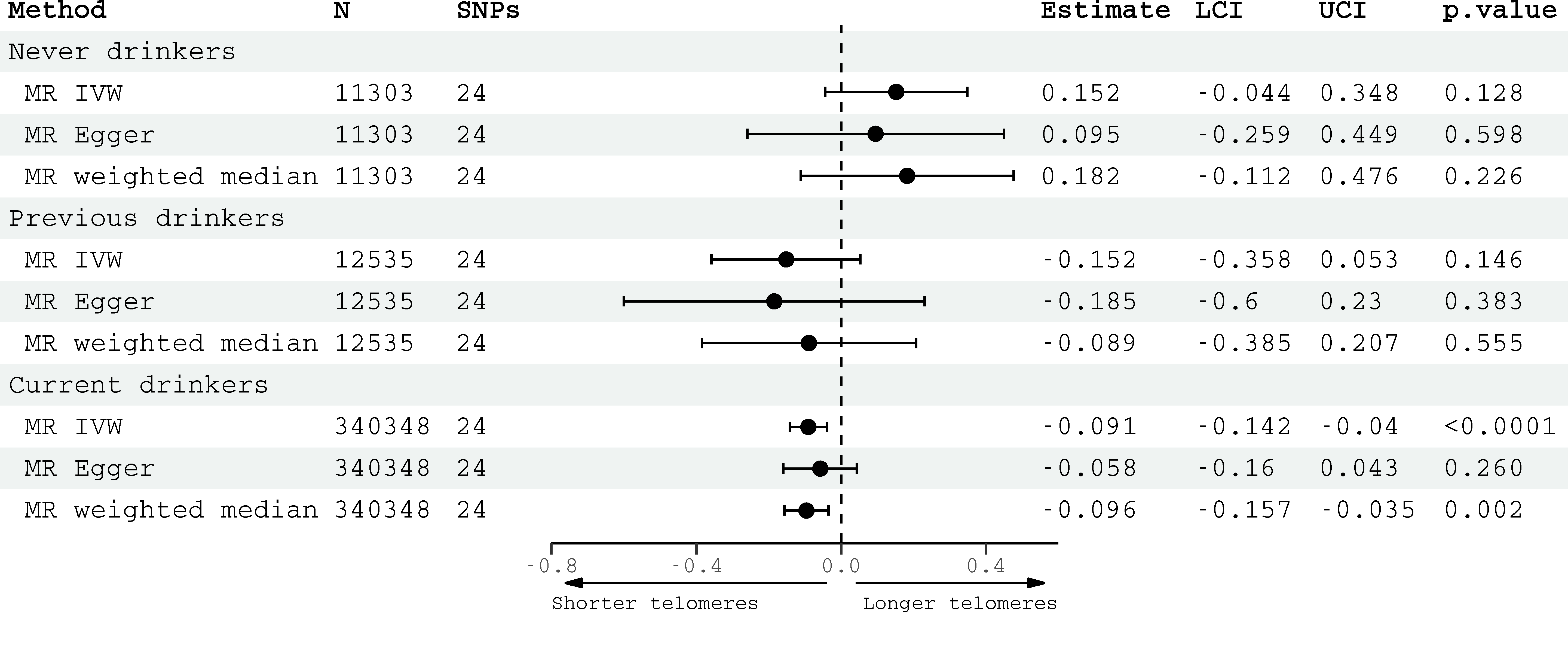
**

**SFigure 18: Negative controls for alcohol use disorder, excluding related participants (kinship coefficient >0.088). Causal estimates for alcohol use disorder on telomere length generated by Mendelian randomization analyses according to alcohol status. Effect estimates represent associations of a genetically-predicted diagnosis of AUD vs. no diagnosis.**
